# Effect of Prenatal Exposure to Polychlorinated Biphenyls on Incidence of Acute Respiratory Infections in Preschool Inuit Children

**DOI:** 10.1289/ehp.8683

**Published:** 2006-03-13

**Authors:** Frédéric Dallaire, Éric Dewailly, Carole Vézina, Gina Muckle, Jean-Philippe Weber, Suzanne Bruneau, Pierre Ayotte

**Affiliations:** 1 Public Health Research Unit, Laval University Medical Center–Centre Hospitalier Universitaire de Québec, Québec City, Québec, Canada; 2 Centre de Toxicologie, Institut National de Santé Publique du Québec, Sainte-Foy, Québec, Canada

**Keywords:** cord blood, environmental health, human, infections, Inuit, organochlorines, pesticides, polychlorinated biphenyls, prenatal exposure, respiratory tract infections

## Abstract

**Objective:**

We set out to assess whether environmental prenatal exposure to polychlorinated biphenyls (PCBs) is associated with incidence of acute respiratory infections in preschool Inuit children.

**Study design:**

We reviewed the medical charts of 343 children from 0 to 5 years of age and evaluated the associations between PCB-153 concentration in umbilical cord plasma and the incidence rates of acute otitis media (AOM) and of upper and lower respiratory tract infections (URTIs and LRTIs, respectively).

**Results:**

The incidence rates of AOM and LRTIs were positively associated with prenatal exposure to PCBs. Compared with children in the first quartile of exposure (least exposed), children in fourth quartile (most exposed) had rate ratios of 1.25 (*p* < 0.001) and 1.40 (*p* < 0.001) for AOM and LRTIs, respectively. There was no association between prenatal PCB exposure and incidence rate of URTIs or hospitalization.

**Conclusion:**

Prenatal exposure to PCBs could be responsible for a significant portion of respiratory infections in children of this population.

It is well known that Inuit children from Canada, United States, and Greenland suffer from a high incidence of respiratory infections, and many authors have identified higher rates of ear infections and lower respiratory tract infections (LRTIs) in Inuit populations compared with Caucasian populations (Banerji et al. 2001; Bluestone 1998; Curns et al. 2002; Davidson et al. 1994; Holman et al. 2001; Karron et al. 1999; Koch et al. 2002; Ling et al. 1969; Lowther et al. 2000; Wainwright 1996). Among the factors suspected to be involved in this phenomenon, perinatal exposure to persistent organic pollutants has been implicated (Dallaire et al. 2004; Dewailly et al. 2000). The immunotoxic potential of some organochlorine compounds (OCs), such as 2,3,7,8-tetrachlorodibenzo-*p*-dioxin (TCDD) and polychlorinated biphenyls (PCBs), is well known (Belles-Isles et al. 2002; Chang et al. 1982; Hoffman et al. 1986; Lu and Wu 1985; Neubert et al. 1992; Tryphonas et al. 1991a, 1991b). Although their production and use are now banned in many countries, a significant proportion of what has been emitted in the environment is still present in the biota of almost every region of the world (Braune et al. 1999; Burkow and Kallenborn 2000; Macdonald et al. 2000). The high degree of chlorination of OCs renders them resistant to biodegradation. They accumulate in adipose tissues of living organisms and are biomagnified in the food chain (Evans et al. 1991). The highest plasma concentrations were observed in top predator species (Braune et al. 1999; Muir et al. 1999; Skaare et al. 2000) and in humans with seafood-rich diets (Bjerregaard et al. 2001; Dewailly et al. 1993; Humphrey et al. 2000; Sjodin et al. 2000).

The Nunavik region is located in the northernmost part of the province of Québec, Canada. Around 9,600 Inuit inhabit 14 Inuit communities spread out on the coastline of Hudson Bay, the Hudson Strait, and the Ungava Bay. For cultural and economical reasons, carnivorous fish and marine mammals constitute an important part of the diet of the Inuit population of Nunavik. Their exposure to food-chain contaminants, such as OCs, is thus proportionally high. Several studies have identified markedly higher concentrations of OCs in adult blood, umbilical cord blood, and breast milk of Nunavik inhabitants, compared with those of the mostly Caucasian southern Québec population (Ayotte et al. 1997, 2003; Dewailly et al. 1993; Muckle et al. 1998, 2001b; Rhainds et al. 1999).

In 2000, we published a first study showing an association between perinatal exposure to OCs and acute otitis media (AOM) in Nunavik Inuit infants (Dewailly et al. 2000). To further document this association, we investigated the relation between maternal OC concentrations and acute respiratory and gastrointestinal infections in a second cohort of 199 infants of the same population (Dallaire et al. 2004). We found that OC concentrations in maternal plasma were positively associated with incidence of acute infections during the first 6 months of life, but not afterward. The number of subjects was small, however, and the associations were not always statistically significant. To clarify the possible link between prenatal exposure to OCs and infections in this population, we report here the association between PCB-153 concentrations in umbilical cord blood and incidence rate of acute respiratory tract infections in a third cohort of 343 preschool children of Nunavik born between 1993 and 1996.

## Materials and Methods

### Study population

Between 1993 and 1996, we monitored the concentrations of OCs and heavy metals in umbilical cord blood of Nunavik newborns (Dewailly et al. 1998). Four hundred ninety-one unselected pregnant women from the 14 Inuit communities of Nunavik were enrolled in the study. The women were invited to participate at their arrival at one of the two health centers in Nunavik for delivery (Puvirnituq and Kuujjuaq). Women giving birth elsewhere were not included. A sample of cord blood was taken, and an interview was conducted with the mothers 1–4 weeks after delivery. When we initiated the present study, children born to these mothers were between 5 and 7 years of age. They were the targeted participants for the present study. The study protocol was reviewed and approved by the Nunavik Health and Nutrition Committee and by the ethics committee of Laval University. All participants gave written informed consent before the study.

### Medical chart review and interview

We attempted to locate and review the medical charts of all the children included in the cord blood monitoring program mentioned above. Five second- and third-year trained medical students reviewed the charts using a standardized questionnaire. For every diagnosis of infection noted in the charts, we recorded the date of diagnosis, whether antibiotics were prescribed, and whether the child was hospitalized. For each infection, we also attributed a code corresponding to the *International Classification of Primary Care* (World Organization of National Colleges, Academies and Academic Associations of General Practitioners 1998). For the present study, we only considered ear and respiratory infections. We formed three categories: upper respiratory tract infections (URTIs), LRTIs, and AOM. Because previous studies on OCs and infections in children seem to point toward a greater association between OCs and otitis media compared with other infectious diseases, we decided to exclude ear infections from the URTI category so that otitis and URTIs could be analyzed independently (Chao et al. 1997; Dewailly et al. 2000; Weisglas-Kuperus et al. 2000). The URTI category included streptococcal pharyngitis and tonsillitis, acute URTI not otherwise specified (NOS), acute rhinitis, head cold, nasopharyngitis, pharyngitis, coryza, sinusitis, tonsillitis NOS, laryngitis NOS, tracheitis, croup, and influenza. In the LRTI category, we included acute bronchitis and bronchiolitis, acute lower respiratory infections NOS, chest infections NOS, laryngotracheobronchitis, tracheobronchitis, bacterial and viral pneumonia, bronchopneumonia, influenzal pneumonia, and pneumonitis. For ear infections, only AOM was included. We excluded otitis media with effusion, chronic otitis media, and glue ears.

We documented perinatal factors using data from the medical charts review and the postpartum interview. These factors were maternal age at parturition, smoking during pregnancy, sex of the child, parity, vaccination, reviewer of the medical chart, and gestational age.

### Determination of OCs in umbilical cord blood

In the original cord blood monitoring program, we determined the concentrations of 14 PCB congeners (International Union of Pure and Applied Chemistry congeners 28, 52, 99, 101, 105, 118, 128, 138, 153, 156, 170, 180, 183, and 187), hexachlorobenzene, and selected chlorinated pesticides and their metabolites [aldrine, α-chlordane, γ-chlordane, *cis*-nonachlor, *p*,*p*′-dichlorodiphenyldichloro-ethylene (DDE), *p*,*p*′-dichlorodiphenyl-trichloroethane (DDT), mirex, oxychlordane, *trans*-nonachlor, and β-hexachlorocyclohexane] in plasma samples by high-resolution gas chromatography. These were singled out because they have been widely used and are the most environmentally persistent. Plasma samples (2 mL) were extracted, cleaned on Florisil columns, taken to a final volume of 100 μL, and analyzed on an HP-5890 series II gas chromatograph equipped with dual-capillary columns and dual Ni-63 electron-capture detectors (Hewlett-Packard, Palo Alto, CA, USA). We identified peaks by their relative retention times obtained on the two columns. The limit of detection was 0.02 μg/L.

### Determination of blood lipids

Because OCs are stored mainly in body fat, all contaminant results are expressed on a lipid basis. We measured total cholesterol, free cholesterol, and triglycerides in plasma samples by standard enzymatic procedures. Phospholipid concentrations were determined according to the enzymatic method of Takayama et al. (1977) using a commercial kit (Wako Pure Chemical Industries, Richmond, VA, USA). We estimated the concentrations of total plasma lipids using the formula developed by Phillips et al. (1989).

### Estimation of prenatal exposure to PCBs

PCB-153 in cord blood was used as a proxy measure for the total PCB burden at birth. PCB-153 is the most abundant congener, and its concentration is strongly correlated with all the moderate to highly chlorinated PCB congeners and with most chlorinated pesticides. For these reasons, it has been shown to be a good marker of exposure to most OCs in the Arctic aquatic food chain (Muckle et al. 2001b). Participants were grouped according to their quartile of PCB-153 concentrations in cord blood. Children in the lowest quartile were used as the group of reference.

### Statistical analyses

Contaminant concentrations had lognormal distributions and were log-transformed in all analyses. Therefore, contaminants results are presented as geometric means. We used Poisson regression to evaluate incidence rate ratio (RR) using the number of diagnosed episodes of infection during the first 5 years of life as the dependent variable and PCB-153 concentration in cord blood as the main independent variable. For every analysis, we constructed two models: one in which exposure to PCB-153 was treated in categories (quartiles of exposure with the lowest quartile as the group of reference), and one in which it was treated in continuous (log transformed). The categorical model yielded estimates of the incidence RRs for infants in the three highest quartiles of exposure, when infants in each quartile are compared with those in the lowest quartile. The continuous model yielded a single RR corresponding to the relative increase in rate for each log increase of the concentration of PCB-153.

We adjusted confounding factors using multiple regression (Poisson regression). Potential confounding variables were tested in the model one by one, but only those influencing the incidence RRs by > 5% were included in the final model. The variables initially excluded were retested one by one in the final model to ensure that their exclusion did not influence the results. The variables included in the final adjusted model were maternal age (10-year categories) and parity (categories). The variables excluded were smoking during pregnancy (yes/no), sex of the child, reviewer of the medical chart, and gestational age (preterm/term). Vaccination coverage was considered as a potential confounding factor, but the information on vaccination gathered through the review of the medical chart was inconsistent. Because preliminary analyses showed that vaccination coverage was not related to contaminant burden, and because we found no scientific report linking vaccination coverage with OC exposure, we excluded it from the final analyses.

We used SPSS Data Entry Builder (version 2.0; SPSS Inc., Chicago, IL, USA) for data entry and SAS (version 8.02; SAS Institute Inc., Cary, NC, USA) for database management and statistical analyses. A *p*-value < 0.05 was considered significant.

## Results

### Participants

Four hundred ninety-one women were included in the initial cord blood monitoring program. Fifty children were initially excluded because contaminant concentrations were not available or because there was not enough information in our database to trace the charts. Of the 441 remaining participants, it was impossible to get the chart of 43 (9.8%) children for various logistical reasons. Among the 398 available charts, 28 (7.0%) were incomplete, 17 (4.3%) families moved out of Nunavik during follow-up, 7 (1.8%) children died, and 3 (0.8%) children were excluded because they suffered from a serious chronic disease. The final analyses included the 343 remaining children. Table 1 shows the characteristics for all participants.

### Contaminant concentrations

Detailed contaminant concentrations in cord blood for these children have been published elsewhere (Dewailly et al. 1998). On a lipid basis, the geometric mean concentration of the sum of the 14 PCB congeners (∑PCBs) in cord blood was 323.5 μg/kg. The PCB-153, the most abundant, had a mean concentration of 93.6 μg/kg. The quartile limits of PCB-153 were Q1, 12.3–58.2 μg/kg; Q2, 58.3–98.3 μg/kg; Q3, 98.4–150.5 μg/kg; and Q4, 150.6–653.6 μg/kg. Based on these quartiles limits, the mean concentrations for the four quartiles of PCBs prenatal exposure were 147.8 μg/kg, 261.8 μg/kg, 395.4 μg/kg, and 708.9 μg/kg for ∑PCBs, and 38.5 μg/kg, 77.7 μg/kg, 120.8 μg/kg, and 229.2 μg/kg for PCB-153.

### Infection incidence rates

The medical chart review of the 343 participants allowed us to identify 5,354 outpatient visits that led to a diagnosis of respiratory infections before 5 years of age. Annualized incidence rates of AOM, URTIs, and LRTIs are shown in Table 2. In children < 2 years of age, AOM was the most frequently diagnosed infection, followed by URTIs and LRTIs. In children ≥ 2 years of age, URTIs were more frequent than AOM. Hospitalizations were frequent: 17.4% of out-patient visits for LRTIs led to an admission. The rate of hospitalizations for LRTIs was 303, 146, and 36 hospitalizations per 1,000 child-years for children 0–11 months, 12–23 months, and 2–5 years of age, respectively.

### Prenatal exposure and AOM

Table 3 presents the association between exposure to PCB-153 and AOM, URTIs, and LRTIs. In the unadjusted model, prenatal exposure to PCB-153 was associated with AOM incidence rates in a dose–response fashion (RRs = 1.13, 1.18, and 1.25 for the second, third, and fourth quartiles, respectively). In the unadjusted continuous model, we observed a 6.5% increase of AOM rates for each log increase of PCB-153 concentration. In the adjusted model, we observed a higher effect size with lower *p*-value compared with that of the unadjusted model.

### Prenatal exposure and URTIs

For URTIs, we did not observe significant associations in either model (Table 3). We observed a weak negative association between URTIs and prenatal exposure to PCB-153, especially for children in the third quartile of exposure. In the unadjusted continuous model, the association was negative, but not statistically significant.

### Prenatal exposure and LRTIs

The highest effect size was seen with LRTIs (Table 3). RRs ranged between 1.21 and 1.40 in the unadjusted model and between 1.25 and 1.44 in the adjusted model. All associations were statistically significant. Although the continuous models were statistically significant, a dose–response pattern was not obvious in the categorical models.

## Discussion

The aim of this study was to identify an association between prenatal exposure to PCBs and rate of acute respiratory infections during the first 5 years of life. We observed that children in the higher quartiles of exposure had a significantly higher incidence rate of outpatient visits for AOM and LRTIs but not for URTIs. This is the third study in which a positive association has been observed between OCs and respiratory infection incidence or prevalence in this population. In a cohort of 98 breast-fed infants < 1 year of age recruited in 1989–1990, we first observed that infants with higher perinatal exposure to OCs through breast-feeding had a higher prevalence of recurrent otitis media compared with that of infants in the lowest exposure group (Dewailly et al. 2000). In a second cohort of 199 infants < 1 year of age, we found that the incidence rates of ear infections and LRTIs were positively associated with PCB-153 and DDE concentration in maternal blood (Dallaire et al. 2004). In the later study, the association was present only during the first 6 months of life. The present study confirms the associations previously observed. Furthermore, it shows that the relation between PCBs and respiratory infections seems to persist past the first months of life.

In the scientific literature, higher rates of respiratory and ear infections have been reported in children born to mothers accidentally or occupationally exposed to PCBs, compared with controls (Chao et al. 1997; Hara 1985; Rogan et al. 1988). For environmental exposure, the evidences of a harmful effect of OCs on infection incidence in children is not yet clear, because both an association (Karmaus et al. 2001; Smith 1984; Weisglas-Kuperus et al. 2000, 2004) and an absence of association (Rogan et al. 1987; Weisglas-Kuperus et al. 1995) have been reported.

Infection incidence rate in children can be affected by several factors, which make the control for confounding difficult. Furthermore, we could not gather information for postnatal factors for most children. Potential postnatal confounding factors such as breast-feeding, household crowding, secondhand smoke exposure, and socioeconomic status have been evaluated in a preliminary study for 90 children from this cohort and did not appreciably affect the associations shown in this study (data not shown). In a previous study from our group assessing the same association between OCs and infections, the several post-natal factors considered in the regression models only slightly increased the effect size of the association (Dallaire et al. 2004). We thus concluded that, in this population, the potential factors that have been considered so far only slightly confounded the association by pulling the RRs toward 1.0. Therefore, unadjusted associations between PCBs and infection rates for this population are likely to be slightly underestimated.

The environmental exposure to OCs for most populations, including the Inuit from Nunavik, consists of a complex mixture of persistent lipophilic chlorinated substances. Because plasma concentrations for most of them are closely correlated with each other (Muckle et al. 2001b), it is impossible to determine which of these compounds—or which combination of them—is responsible for the association. In our previous study, DDE concentration in maternal plasma was found to be more closely associated with infection incidence rates compared with PCB-153 concentration (Dallaire et al. 2004). In the present study, results for DDE exposure are not presented but were in general similar to those for PCB-153. Although our analyses were conducted using PCB-153 as a proxy for OC exposure, the potential harmful effect on the immune system could be attributed to other compounds highly correlated to PCB-153 concentration.

The associations shown in this study were estimated using prenatal exposure only. Although the immune system is most vulnerable during its development *in utero*, postnatal exposure to the same compounds through breast-feeding and food consumption could also increase susceptibility to infections. It is likely that prenatal and postnatal exposures were correlated because eating habits of a mother will probably influence her child’s diet. It is therefore possible that part of the association with prenatal exposure was actually due to postnatal exposure.

In this study, we used a review of the medical charts to evaluate incidence rates. There is only one health center in each community included in this study. Participants almost always visit that health center when they seek medical attention, and copies of consultations done elsewhere are routinely requested to complete medical charts. We are therefore confident that we have reviewed most outpatient visits sought by the participants. Nevertheless, we did not attempt to verify every diagnosis, nor did we try to inquire about infections for which medical attention was not sought by the parents. It is therefore important to keep in mind that the incidence rates reported here are underestimated. We cannot exclude the possibility that the propensity to seek medical attention when respiratory symptoms are present was associated with traditional lifestyle, which in turn is known to be associated with OC concentration in maternal blood (Muckle et al. 2001a). Should this happen with our participants, the direction of the bias that would be introduced would be unknown. We find it improbable, however, that Inuit families with a traditional lifestyle would increase their frequency of medical contacts in such a way that the full extent of the observed association would be solely due to this bias, if any.

Inuit children from Nunavik are burdened by a high rate of respiratory infectious diseases. In a related study on infection incidence conducted with the same cohort, we showed that LRTIs are far more frequent in Nunavik compared with other Canadian populations and that the hospitalization rate for LRTIs in Nunavik was one of the highest ever reported in recent scientific literature (Dallaire et al., in press). If the association between respiratory infection and prenatal exposure to PCBs observed in this population is causal, exposure to PCBs during development would be responsible for a clinically significant proportion of respiratory infectious episodes in these children. The biologic mechanism of this effect in humans environmentally exposed is still obscure. Other studies are needed to identify which immune pathways are affected in exposed children.

## Figures and Tables

**Table 1:** Characteristics of participants (*n* = 343).

Characteristic	Value
Children	
Male sex (%)	49.0
Year of birth (%)	
1994	37.3
1995	32.1
1996	30.6
Hospital of delivery (%)	
Puvirnituq	48.7
Kuujjuaq	51.3
Gestational age (weeks)	
Mean gestational age	39.1
Preterm [< 37 weeks (%)]	5.3
Birth weight [mean (g)]	3,494
Length [mean (cm)]	51.5
Mothers	
Age [mean (years)]	23.7
Parity (mean)	2.1

**Table 2:** Incidence rate of respiratory infections during the first 5 years of life.

	Incidence rate (95% CI) by age: events per 1,000 child-years		
Infection	< 12 months	12–23 months	24–60 months
All respiratory infections	5,434 (5,066–5,803)	4,466 (4,119–4,814)	1,908 (1,732–2,083)
AOM	2,128 (1,932–2,324)	2,087 (1,885–2,290)	697 (614–779)
URTIs	1,974 (1,799–2,149)	1,487 (1,338–1,636)	888 (786–991)
LRTIs	1,332 (1,171–1,493)	892 (769–1,015)	323 (276–369)

CI, confidence interval.

**Table 3:** Incidence RR of AOM, URTIs, and LRTIs according to prenatal exposure to PCB-153.

	RR (95% CI)		
Prenatal exposure model	AOM	URTIs	LRTIs
Unadjusted model (*n* = 343)			
Continuous (for each log increase)	1.065 (1.002–1.131)*	0.943 (0.887–1.002)	1.109 (1.019–1.208)*
Categories^a^			
Q1 (least exposed)	1.00 (referent)	1.00 (referent)	1.00 (referent)
Q2	1.13 (1.00–1.28)*	0.96 (0.86–1.08)	1.37 (1.15–1.63)*
Q3	1.18 (1.04–1.33)*	0.90 (0.80–1.02)	1.21 (1.01–1.44)*
Q4 (most exposed)	1.25 (1.10–1.41)*	1.00 (0.89–1.12)	1.40 (1.18–1.67)*
Adjusted model (*n* = 330)			
Continuous (for each log increase)	1.123 (1.052–1.199)*	0.995 (0.931–1.063)	1.135 (1.036–1.243)*
Categories^a^			
Q1 (least exposed)	1.00 (referent)	1.00 (referent)	1.00 (referent)
Q2	1.15 (1.01–1.31)*	0.99 (0.87–1.12)	1.39 (1.16–1.66)*
Q3	1.26 (1.11–1.43)*	0.95 (0.83–1.07)	1.25 (1.04–1.50)*
Q4 (most exposed)	1.37 (1.20–1.55)*	1.09 (0.97–1.24)	1.44 (1.20–1.72)*

CI, confidence interval.

a Quartiles of PCB-153 concentration in cord blood.

* *p* < 0.05.
